# Intensity of physical activity as a percentage of peak oxygen uptake, heart rate and Borg RPE in motor-complete para- and tetraplegia

**DOI:** 10.1371/journal.pone.0222542

**Published:** 2019-12-03

**Authors:** Tobias Holmlund, Elin Ekblom-Bak, Erika Franzén, Claes Hultling, Kerstin Wahman

**Affiliations:** 1 Department of Neurobiology, Care Sciences and Society, Division of Neurogeriatrics, Karolinska Institutet, Stockholm, Sweden; 2 Rehab Station Stockholm/Spinalis R&D Unit, Solna, Sweden; 3 Åstrand Laboratory of Work Physiology, The Swedish School of Sport and Health Sciences, Stockholm, Sweden; 4 Department of Neurobiology, Care Sciences and Society, Division of Physiotherapy, Karolinska Institutet, Stockholm, Sweden; 5 Allied Health Professionals Function, Function Area Occupational Therapy & Physiotherapy, Karolinska University Hospital, Stockholm, Sweden; 6 Spinals Foundation–R&D Unit, Stockholm, Sweden; Universitat Konstanz, GERMANY

## Abstract

**Objective:**

The aims were to describe VO_2peak_, explore the potential influence of anthropometrics, demographics and level of physical activity within each cohort; b) to define common, standardized activities as percentages of VO_2peak_ and categorize these as light, moderate and vigorous intensity levels according to present classification systems, and c) to explore how clinically accessible methods such as heart-rate monitoring and Borg rating of perceived exertion (RPE) correlate or can describe light, moderate and vigorous intensity levels.

**Design:**

Cross sectional.

**Setting:**

Rehabilitation facility and laboratory environment.

**Subjects:**

Sixty-three individuals, thirty-seven (10 women) with motor-complete paraplegia (MCP), T7-T12, and twenty-six (7 women) with motor-complete tetraplegia (MCT), C5-C8.

**Interventions:**

VO_2peak_ was obtained during a graded peak test until exhaustion, and oxygen uptake during eleven different activities was assessed and categorized using indirect calorimetry.

**Main outcome measures:**

VO_2peak_, Absolute and relative oxygen consumption, Borg RPE.

**Results:**

Absolute VO_2peak_ was significantly higher in men than in women for both groups, with fairly small differences in relative VO_2peak_. For MCP sex, weight and time spent in vigorous-intensity activity explained 63% of VO_2peak_ variance. For MCT sex and time in vigorous-intensity activity explained 55% of the variance. Moderate intensity corresponds to 61–72% HR_peak_ and RPE 10–13 for MCP vs. 71–79% HR_peak_, RPE 13–14 for MCT.

**Conclusion:**

Using current classification systems, eleven commonly performed activities were categorized in relative intensity terms, (light, moderate and vigorous) based on percent of VO_2peak_, HR_peak_ and Borg RPE. This categorization enables clinicians to better guide persons with SCI to meet required physical activity levels.

## Introduction

Physical activity (PA) is important in spinal cord injury (SCI) rehabilitation for improving physical function, to increase energy expenditure, but also for the prevention and treatment of the increasingly prevalent cardiovascular risk factors[1–6]. Despite this, PA level and physical fitness are generally low in SCI populations compared to the general population[7, 8]. Therefore, it’s essential for individuals with SCI to be more physically active. To facilitate this, it is of interest to define intensity levels as relative values and to describe a variety of common physical activities by intensity level. The intensity of an activity is, especially in populations with low VO_2peak_ consumption[9], best described in relation to peak capacity, often expressed as a percentage of oxygen consumption and heart rate at peak effort (here defined as VO_2peak_ and HR_peak_, respectively), or percentage of HR_peak_ reserve. Rating of perceived exertion according to the Borg RPE-scale is another common and easily accessible method[10]. The perceived intensity and percentage of VO_2peak_ of activity (e.g. wheelchair wheeling at 5 km/h or ergometer arm cycling at 50 watts) constitute one of the variables closely connected to an individual’s VO_2peak_, and this may vary widely between individuals.

However, it is hard in clinical practice to assess VO_2peak_ or HR_peak_^.^ This makes it difficult to assess relative intensity level accurately in people with an SCI, especially motor-complete tetraplegia[11]. Previous studies have reported intensity levels according to the Borg RPE scale and percentage of HR_peak_ from arm-ergometer VO_2peak_ tests[12–14]. Other studies have used percentage of HR_peak_ to describe time spent at different intensity levels during rehabilitation[7, 15]. More easily accessible methods to describe relative intensity levels would facilitate clinical work and allow better guidance towards a physically active lifestyle. Moreover, there are one extensive studie that have collected data from standardized activities and used SCI METs[16]; however the study didn’t relate the VO_2_ to peak or to Borg RPE or HR to describe intensity levels.

The present study of two defined cohorts of persons with tetraplegia (C5-C8) AIS A-B and paraplegia (T7-T12) AIS A-B, aimed a) to describe peak oxygen uptake (VO_2peak_) and explore the potential influence of anthropometrics, demographics and level of physical activity within each cohort; b) to define common, standardized activities as percentages of VO_2peak_ and categorize these as light, moderate and vigorous intensity levels according to present classification systems, and c) to explore/describe how clinically accessible methods such as heart-rate monitoring and rating of perceived exertion correlate, or can describe light, moderate and vigorous intensity levels.

## Methods

### Participants

We studied a convenience sample including 63 persons with SCI; 37 (27 men and 10 women) with motor-complete paraplegia (MCP) AIS A-B (T7–T12), and 26 persons (19 men and 7 women) with motor-complete tetraplegia (MCT) AIS A-B (C5–C8). All were recruited through SCI-specific websites or by word of mouth. Inclusion criteria were SCI injury level C5–C8 and T7–T12; AIS A and B motor-complete, ≥1 year post-injury, age ≥18 years, with minimal spasticity (Baclofen treated), as reported on the spasm frequency scale (Penn)[17]. All participants used manual wheelchairs except for one who used power-assisted wheels indoors the same participant did none of the outdoor activities due to the rain. Exclusion criteria were coronary artery disease, angina pectoris and chronic congestive heart failure, chronic obstructive pulmonary disease, or shoulder pain. Persons were excluded if they were on pharmaceutical treatment such as beta blockers or hormone replacement therapy (thyroid hormone). However, those on Baclofen were included. All data were collected in a rehabilitation setting. The participants were asked to avoid heavy exercise 12 hours before the testing, to refrain from coffee and nicotine and to empty the bladder before testing. Each provided written informed consent, and ethical approval was given by the Stockholm region ethics committee, reference number 2011/1989-31/1. We choose to not include persons with an injury level between T1-T6, to have a clear difference between MCP with sufficient ANS functioning versus MCT with reduced or lack of response from ANS. This is based on that the group T1-T6 produces fuzzy results on HR due to shady differences in ANS function, whereas persons with injury level below T7 have an almost normal functioning cardiovascular response from ANS[18, 19].

### Assessment of VO_2_ during the standardized activities

The VO_2_ assessment procedure has been described previously[20, 21]. In brief, VO_2_ was assessed using the same mobile system as for the VO_2peak_ test. The system was calibrated using built-in automated procedures. All data were collected the same day (except for seven participants who did the VO_2peak_ test on a different day). The sedentary activities included television-watching and desk-based computer work; light intensity included setting a table following a standardized procedure, wheeling a manual wheelchair indoors (in a training hall, wooden floor, 25-meter track with two turns) at their own individual pace perceived as 10–11 (light exertion) on the Borg RPE scale, and individually-paced wheelchair wheeling outside on asphalt also perceived as Borg 10–11. The exercise activities included wheeling the wheelchair outside on asphalt at exercise pace Borg 13–14. Arm ergometer work at 60 rpm was performed at low level (10W or 15W for MCT and 18W or 24W for MCP) and high level (20W or 25W for MCT and 36W or 42W for MCP). For weight training, the instruction was to select a weight that the participant was able to lift ten times, comfortably at an even and controlled pace. This was tested/practiced before data collection began. The participants rested 15–45 seconds between each machine (including transfer and set-up times to the next gym machine). Circuit-resistance training was performed by one second for the concentric phase and two seconds for the eccentric phase, (rowing machine, pulldowns, Pec Dec for MCP and, rowing machine, pulldowns, external, internal rotation for MCT)[22]. The VO_2peak_ tests were performed as the last activity of the day.

Information about each activity was given as a standardized verbal instruction together with (Borg RPE 10–11 for light and 13–14 for moderate) and weight selection in weight training. Each test lasted for 6–7 minutes, and the time between each activity varied between a few minutes (sedentary) and >30 minutes (exercise activities). Before testing started the tire pressure was checked to be between 7–10 BAR depending on manufacturer.

### Assessment of VO_2peak_

A Monark arm ergometer (Ergomedic 891E Monark, Sweden) was used for the VO_2peak_ test. It was attached to a height-adjustable and the participants were seated in their own wheelchair. Prior to the test, placement and positioning of table and arm ergometer were individually adjusted. Individuals with poor hand function brought their own gloves to be able to hold on to the handlebars. All were asked if they wanted to be strapped to the wheelchair to retain upper-body balance. The test began with approximately three minutes of warm-up, followed by a short break, and was then incremented until exhaustion[23, 24]. The protocol used to achieve VO_2peak_ was individually designed according to international laboratory procedures and previous studies[24] with self-paced cadence starting between 70 and 90 revolutions per minute (rpm) and finishing around 100 to 120 rpm[25]. The starting resistance was chosen depending on level of injury and exercise status based on the resistance during the “low” and “high” arm ergometer activity”, which were performed at the beginning of the test-day. The resistance was subsequently increased every minute by 0.25kg for MCT subjects with low (10W or 15W) resistance during arm ergometer work and 0.5kg for those with high (20W or 25W) resistance. For MCP participants the resistance was increased by 0.5kg for those with 36W resistance during arm ergometer exercise and by 0.75kg for those with 42W. The increase during the last 2–3 minutes was individually managed according to the participant’s state of exhaustion as evaluated from visual/auditory contact. This gave an anticipated time to exhaustion of between 6 and 12 min. VO_2_ and HR were measured continuously during the test using a mobile open-circuit system (Jaeger Oxycon Mobile system (Hoechberg, Germany). VO_2_ was analyzed as the average of 10-second averages. VO_2peak_ was determined as the mean of the highest 30 seconds. Criteria for acceptance of the VO_2peak_ measurement were: “levelling off” of VO_2_ despite increased resistance, RPE above 16, test time more than 6 minutes, supported by a respiratory quotient (RQ/RER) greater than 1.1. All participants reached the criteria of acceptance. None of the participants wore leg wraps and/or abdominal binders.

### Other measurements

Body weight was measured to the nearest 0.1 kg, and height was self-reported, with subsequent calculation of $BMI=\frac{Weight\;(kg)}{Height\;(cm)2}$. Self-reported PA was assessed using a validated questionnaire[26], where time spent in moderate and vigorous PA was reported and subsequently calculated by multiplying the minutes (reported as 15 min bouts) each person spent in moderate, vigorous and leisure-time activity (LTA) by the number of days per week. The questionnaire was further dichotomized according to current SCI guidelines for cardiorespiratory fitness (0–44 or 45–450 minutes per week)[27]. Heart rate was measured with chest-strap (Polar) connected to the Oxycon Mobile.

### Statistical analysis

Statistical analysis used SPSS (SPSS for Windows Version 23.0; Inc. Chicago, IL, USA). All data was tested for normality using Shapiro-Wilk tests and Q–Q plot analyses. For descriptive statistics, mean, SD or median and range were used. An independent sample two-tailed *t*-test for group comparisons and statistical significance was set at α = 0.05. Non-normally-distributed data is presented as median interquartile range, Q1–Q3 (IRQ). Categorical data was analyzed with the Mann-Whitney *U* test. Spearman’s *rho* correlation coefficient was assessed to describe the association between VO_2peak_ and anthropometrics (body weight and height), demographics (age, level of injury and gender) and PA questionnaire (level, duration and intensity), within the cohorts. Further, stepwise (both forward and backward) multiple linear regressions were used to identify factors that could explain variance in VO_2peak_ within each cohort, since VO_2peak_ on group level was normally distributed. The probability for entry into the model was set to F value = p<0.05 and the probability for removal was F value = >0.10. The collinearity statistics variance inflation factor (VIF) was 1.0–1.53 and the variables’ correlation to each other was below 0.6, with a correlation to VO_2peak_ above 0.48. All data for percentage of VO_2peak_ were stratified into standardized levels of activity according to ACSM[9, 28]. The levels for MCP were *light* (37–45%VO_2peak_), *moderate* (46–63% VO_2peak_) and *vigorous* (64–91%VO_2peak_) [9, 28, 29]. The levels for MCT were *light* (44–51% VO_2peak_), *moderate* (52–67%VO_2peak_) and *vigorous* (68–94%VO_2peak_). For an activity to be categorized into an intensity all variables, % of VO_2peak_, Borg RPE, % of HR_peak_ and speed (kmh), were analyzed and median and interquartile range was used to classify each activity. The MET-value for VO_2peak_, calculated with help of the individual REE from the same cohort, previously published by our research group, shows a MET ≈8 for MCP and ≈5 for MCT[20]. Categorization of the activities was made by examining how many persons (in each activity) that were categorized by stratifying for % of VO_2peak_ Borg RPE and HR. At least 66% of the participants in each activity needed to be categorized correct.

## Results

The study participants’ characteristics showed significant gender differences for height, weight and BMI both for persons with MCP and for those with MCT (Table 1).

**Table 1:** Characteristics of study participants and heart rate and oxygen consumption during rest and peak oxygen consumption.

		*Tetraplegia n = 26*^*a*^			Paraplegia n = 37	
	Mean ± SD (Median/IQR)			Mean ± SD (Median/IQR)		
	*all*	*men (n = 19)*	*women (n = 7)*		*all*	*men (n = 27)*	*women (n = 10)*
Age (years)	41.5±14.0	41.2±14.5	42.4±11.9	42.7±11.4	44.1±11.6	38.8±10.8
Height (cm)	178±0.09	181±0.08^b^	1.68±0.05	177±0.10	181±0.08^b^	165±0.05
Weight (kg)	65.3±12.9^c^	70.1±11^b^	52.3±7.41	72.9±15.1	77.8±13.2^b^	59.7±8.5
BMI	20.5±3.0^c^	21.3±2.93^b^	18.4±1.94	23.1±3.3	23.6±3.30	21.9±3.28
Years since injury	15.3±10.9	15.2±11.8	15.4±8.73	15.6±11.4	15.9±11.8	14.6±10.8
RMR HR	(47.2/44-53)	(48/45-52)	(46.2/39-59)	(61/53-69)	(56/48-64)	68±3.3
RMR VO2 (L∙min^-1^)	0.16±0.03^a^	0.17±0.03^*a*^	0.14±0.02^a^	0.18±0.04^a^	0.19±0.03^*a*^	0.15±0.03^*a*^^b^
HR_peak_	(108/97-119)	(108/99-122)	(109/94-115)	(176/164-188)	(177/163-187)	(174/168-189)
VO_2peak_ (L∙min^-1^)	(0.74/0.60–0.89)	(0.84/67-98)	(0.53/45-65)	(1.36/1.14–1.65)	(1.57/1.26–1.79)	(1.02/0.85–1.14)

BMI = body mass index, cm = centimeter. Kg = kilogram

HR = Herat rate beats/min^-1^

RMR = resting metabolic rate

IQR = inter quartile range

^a^Mean values published in Spinal Cord 2017

^b^Significant difference between men and women

^c^Significant difference between tetraplegia and paraplegia *p*<0.05

### VO_2peak_

**Fig 1:**
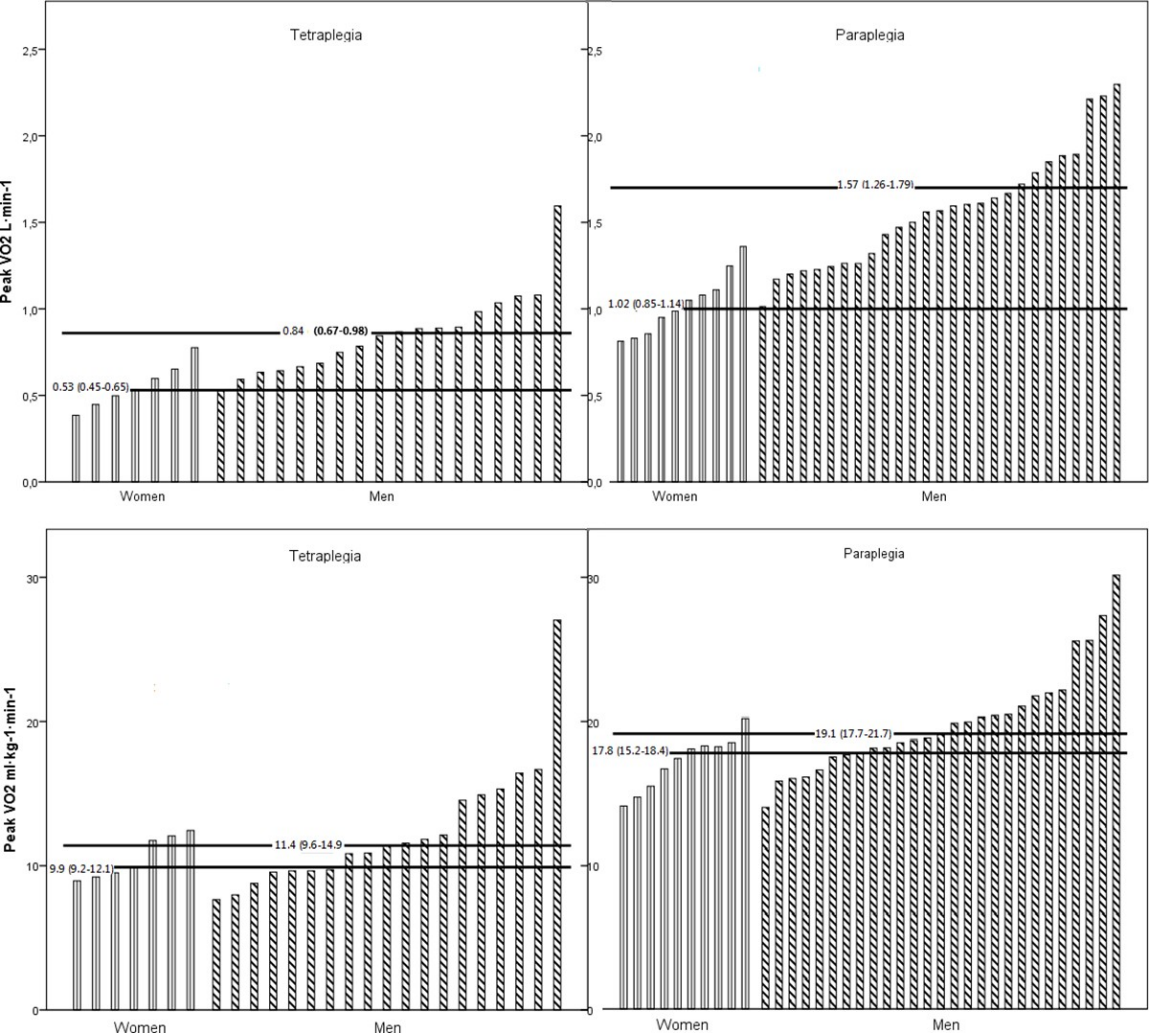
a. Individual absolute VO_2peak_ L·min-1 (IQR) values and group median for tetraplegic (left) and paraplegic (right) women and men. b. Individual relative VO_2peak_ (ml·min-1·kg-1) values and group median for tetraplegic (left) and paraplegic (right) women and men. * Significant difference between men/women, p&<0.002 ** Significant difference between men/women, p&<0.03.

The median for absolute VO_2peak_ for the persons with MCP was 1.36 L·min^-1^ (IQR 1.14–1.65) and 0.74 L·min^-1^ (IQR 0.60–0.89) for those with MCT (Table 1 and Fig 1A). Both men with MCP and MCT had significant higher VO_2peak_ than women, *p*≤0.001. Men with MCP had a VO_2peak_ of 1.57 L·min^-1^ vs. women 1.02 L·min^-1^ (54% higher) and men with MCT had a VO_2peak_ of 0.84 L·min^-1^ vs. woman 0.53 L·min^-1^ (53% higher). Relative VO_2peak_ was 18.5 ml·min^-1^·kg^-1^ (IQR 17.0–20.4) for MCP and 11.1 ml·min^-1^·kg^-1^ (IQR 9.6–13.5) for persons with MCT (Table 1 and Fig 1B). Men with MCP in general had a 7% higher relative VO_2peak_ than women *p =* 0.02, while there was no significant gender difference within the MCT group *p* = 0.43. Gross mechanical efficiency during submaximal arm-ergometer was ≈8% for 10-15W and ≈13% for 20-25W (MCT) and for MCP ≈10% (18-24W) and ≈14% 24-36W).

### Relationship between VO_2peak_ and anthropometric, demographic and PA variables

Persons with MCP that were physically active at vigorous intensity level at least 45 min per week had a significantly higher VO_2peak_ 1.7 L·min^-1^ compared to 1.3 L·min^-1^ *p* = 0.007. For persons with MCT, the difference was not significant: 0.87 L·min^-1^ (more than 45 mins/week) vs. 0.68 L·min^-1^ (p = 0.02).

After controlling for ten different variables (Table 2) sex, body weight, body height and self-reported time spent in vigorous activity per week showed a significant weak- (r = 0.40 to 0.59)-to-moderate (r = 0.60 to 0.79) correlation[30] with VO_2peak_ for both cohorts. These four variables were further introduced into a stepwise regression analysis that revealed that sex (b = 0.26, p = 0.02), weight (b = 0.01, p<0.001) and time spent in vigorous intensity activity (b = 0.001, p = 0.03) explained 63% (R^2^ = 0.63) of the VO_2peak_ variance in MCP. For MCT, sex (b = 0.20, p = 0.02) and time spent in vigorous intensity activity (b = 0.001, p = 0.001) explained 55% of the variance (R^2^ = 0.551).

**Table 2:** Association between different demographical, anthropometric and physical activity variables with VO_2peak_.

** **	**Paraplegia**	**p-value**	**Tetraplegia**	**p-value**
**Variable**	Correlations coefficient (r)		Correlations coefficient (r)	
**Sex**	0.67	<0.001	0.60	0.001
**Age**	0.02	0.888	-0.22	0.293
**BMI**	0.43	0.008	0.29	0.156
**Body weight**	0.74	0.001	0.57	0.003
**Body height**	0.68	<0.001	0.56	0.003
**Level of injury**	0.22	0.199	0.29	0,379
**Time since injury**	0.07	0.661	-0.35	0.087
**PA Questionnaire (min/week)**	0.30	0.067	0.06	0.773
**Leisure time activity light**				
**Moderate intensity (min/week)**	0.22	0.203	0.49	0.015
**Vigorous intensity (min/week)**	0.35	0.033	0.46	0.025

BMI = body mass index. Min = minutes. PA = physical activity

### Standardized activities as percentages of VO_2peak_

**Fig 2:**
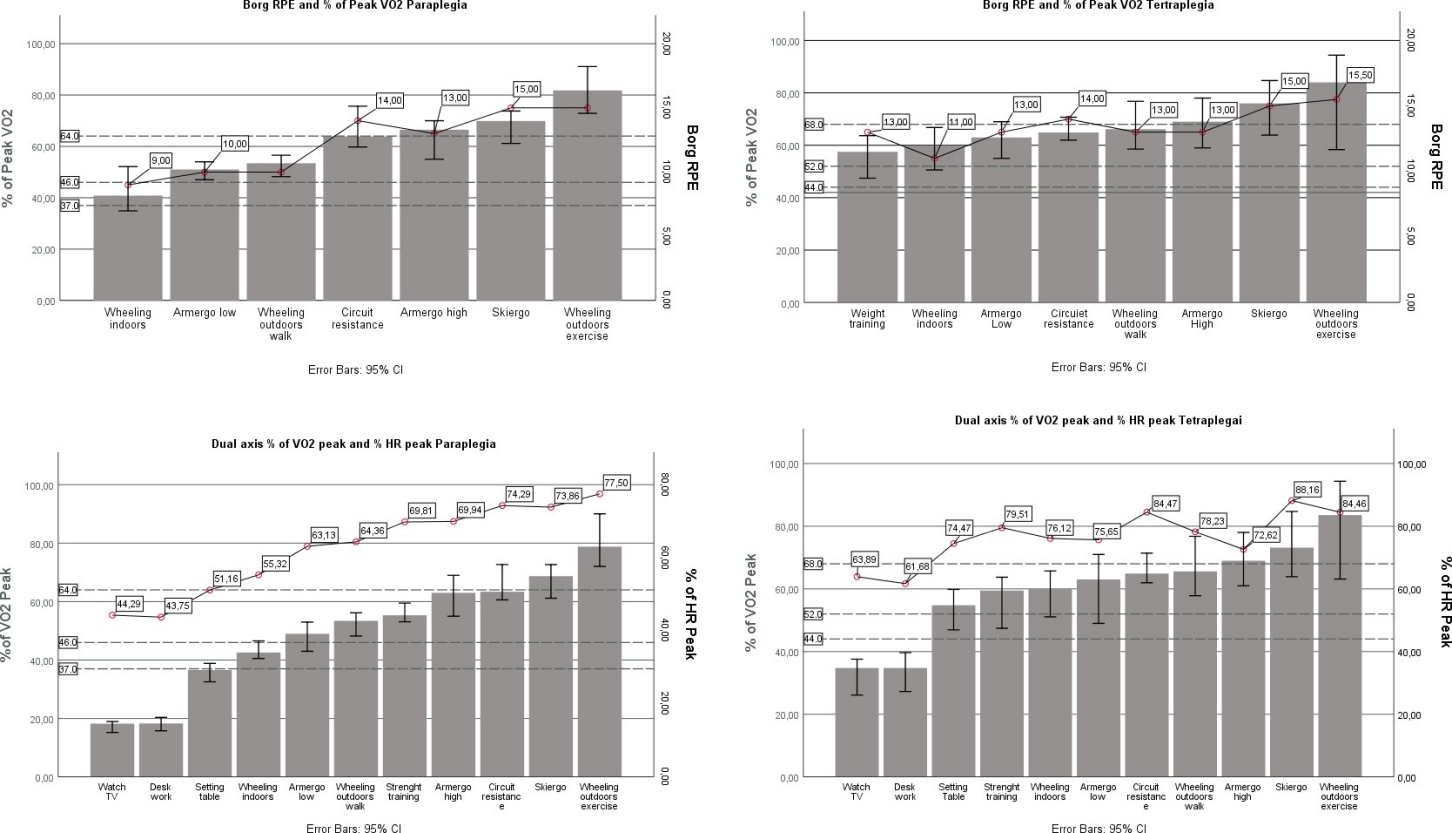
Dual axis for VO_2peak_ (mean 95% CI) and Borg RPE or % of HR_peak_ during activities’, for MCT/MCP. Wheeling indoors (Borg 10–11) Km h^-1^, mean (range). Paraplegia 4.4 (2.9–5.8). Tetraplegia 4.2 (2.0–6.7). Wheeling outdoors (Borg 10–11) Km h^-1^, mean (range). Paraplegia 6.6 (4.7–8.5). Tetraplegia 5.2 (1.9–7.4). Wheeling outdoors (Borg 13–14) Km h^-1^, mean (range). Paraplegia 8.9 (6.3–12.0). Tetraplegia 7.6 (5.5–8.6).

The categorization of the eleven activities into different intensity levels, expressed as % of VO_2peak_, Borg RPE (seven activities) and % of HR_peak_ (Fig 2). Some activities in Fig 2 have a 95% CI within two intensity levels (wheeling indoors, setting table for MCP), which makes them harder to categorize. However, wheeling indoors could be stratified by speed (kmh), which is done in Table 3 and the heart rate for setting table was 51% HR_peak_ which was categorized as light intensity (Fig 2). Moreover, for MCT setting a table and strength training were within the limits for moderate intensity and wheeling indoors and the other non-exercise activities was categorized between moderate and vigorous (Fig 2). Vigorous intensity included all exercise activities for MCP and MCT.

**Table 3:** Eleven activities categorized into intensity level based on the result for percentage of VO_2peak_, Borg RPE and percentage of HR_peak_.

**Level of intensity**	**Relative intensity Fyss 2015**^**23**^. **ACSM**^**24**^	**Relative intensity Paraplegia Holmlund et al.,**	**Activities, Paraplegia**	**Relative intensity tetraplegia Holmlund et al.,**	**Activities, Tetraplegia**
**Sedentary**	< 37% VO_2max_ < 57% max HR RPE <8	< 37% VO_2peak_ < 50% HR_peak_ RPE <8	▪Watch TV ▪Desk work	< 44% VO_2peak_ < 64% HR_peak_ RPE <9	▪Watch TV ▪Desk work
**Light intensity**	37–45% VO_2max_ 57–63% max HR RPE 8–11	37–45% VO_2peak_ 51–60% HR_peak_ RPE 8–9	▪Setting table ▪Wheeling indoors (3.7–4.7 kmh) ▪Arm-crank 18W	45–51% VO_2peak_ 65–70% HR_peak_ RPE 10–12	
**Moderate intensity**	46–63% VO_2max_ 64–76% max HR RPE 12–13	46–63% VO_2peak_ 61–72% HR_peak_ RPE 10-^(12^*^)^13	▪Wheeling indoors (4.8 ▪-5.8kmh) ▪Wheeling outdoors 4.7-6kmh “walk” ▪Arm-crank 24 W, 36W ▪Weight training	52–67% VO_2peak_ 71–79% HR_peak_ RPE 13–14	▪Setting table ▪Weight training ▪Wheeling indoors 2.0–6.7kmh ▪Arm-crank 10-15W ▪Wheeling outdoors “walk” 2–6 kmh
**Vigorous intensity**	64–90% VO_2max_ 77–94% max HR RPE 14–17	64–90% VO_2peak_ 73–90% HR_peak_ RPE 14–17	▪Arm crank 48W ▪Wheeling outdoors 6.1-12kmh “exercise” ▪Circuit resistance ▪Ski-ergo ▪	68–94% VO_2peak_ 80–89% HR_peak_ RPE 15–17	▪Wheeling outdoors exercise (>5kmh) ▪Circuit resistance ▪Ski ergo ▪Arm crank (20, 25W)

VO_2_ = Oxygen consumption. HR = Heart rate. RPE = Rate of perceived exertion. W = Watt

* = Recommendation for moderate intensity 12–13 Borg RPE

Borg RPE correlated strongly with %VO_2peak_ (*correlation coefficient* = 0.59, *p*<0.001) in MCP and significantly but weakly in MCT (*correlation coefficient* = 0.32, *p*<0.001). The Borg RPE for light intensity had a median 9 (IQR 8–10) for persons with MCP and a median of 12 (IQR 10–13) for MCT. For moderate intensity the Borg RPE median was 10.5 (9.5–13 IRQ) for MCP, and 13 (12–14 IQR) for MCT. For vigorous intensity the Borg RPE median was 14 (13–15 IQR) for MCP, and 14 (13–15 IQR) for MCT (Fig 2). Further, % HR_peak_ correlated strongly to %VO_2peak_ (*correlation coefficient* = 0.79, p<0.001) for MCP and (*correlation coefficient* = 0.63, p<0.001) for MCT. There were no significant differences for HR_peak_ between men and women within the group of persons with MCT and group of persons with MCP. Hence, there was a significant difference between men and women in resting HR among individuals with MCP (Table 1). Percent of HR_peak_ for light intensity showed a median of 52% (46–59 IQR) for MCP versus 74% (66–78 IQR) for MCT. The moderate intensity median was 65% (58–75 IQR) for MCP and 79% (71–85 IQR) for MCT. Vigorous intensity showed a median for relative HR of 75% (68–84 IQR) for MCP and 84% (75–88 IQR) for MCT (Fig 2).

## Discussion

Men were observed to have a significantly higher absolute VO_2peak_ than women while there was a smaller gender difference for relative oxygen consumption. Sex, weight and time spent in vigorous intensity activity explained 63% of VO_2peak_ variance for MCP. For MCT, sex and time in vigorous intensity activity explained 55% of this variance. Moreover, all of the activities of daily life were categorized as moderate-to-vigorous for MCT. Translation of the main results into the categorization scheme (Table 3) is highly clinically relevant, and may function as a tool for choosing activities at certain intensity by combining the activity, RPE and/or % of HR_peak_.

### VO_2Peak_

As in previous literature[31, 32], our results show differences in absolute (L·min^-1^) and relative (ml·min^-1^·kg^-1^) VO_2peak_ between MCP and MCT. This is most likely related to the difference in cardiorespiratory response from the autonomic nervous system[13, 33] and the individual’s functional muscle volume. The results for absolute VO_2peak_ are comparable to previous results for persons with similar levels of injury and completeness[32, 34–39]. Hence our results are lower compared to most male athletes in Olympic sitting disciplines except for shooting[39]. We choose to use arm-ergometer for VO_2peak_ testing. Recent research suggests no difference in VO_2peak_ between arm ergometer peak testing and wheelchair tests for individuals with SCI (T3 –L1 and completeness AIS A-C)[36, 40–43]. Asynchronous arm ergometer was used during testing and has been reported to be produce equal or higher values compared to synchronous during VO_2peak_ testing[44]. Regardless of method, these two assessments differ from the traditional VO_2peak_ for the general population, which tests cardiac output. Individuals with MCP and MCT have limited muscle mass, so these tests assess aerobic capacity to a higher degree[36]. Moreover, the loss of descending sympathetic control is not always equal to the lesion level in MCT as shown when comparing high-performance athletes to non-athletes[11].

### Intensity of standardized activities

This study presents data on the intensity of different standardized activities for persons with MCP and those with MCT, expressed as % of VO_2peak_, % of HR_peak_ and self-reported perceived exertion. Further, in Table 3 we categorize activities based on % of VO_2peak,_ % HR_peak_ and Borg RPE into intensity levels (sedentary, light, moderate and vigorous). Our results are based on general physiological parameters [29, 45] and show that we slightly altered Borg RPE and % HR_peak_ for both groups. The results for % HR_peak_ for persons with MCP are comparable to those in previous studies, where 40% of VO_2peak_ corresponded to 61–66% HR_peak_[13, 46] compared to the present ≈59% HR_peak_. Likewise, 60% of VO_2peak_ corresponded to 73–77% HR_peak_[13, 46] compared to ≈72% HR_peak_ in our study. For MCT, relative HR at moderate and vigorous intensity is comparable to that in previous studies[47, 48]. However, it’s difficult categorize relative HR for MCT due to attenuated heart rate responses due to impairment in descending sympathetic control. However, it’s still of interest to describe the HR for MCT on the basis of the recommended intensity levels from American college of sports medicine[9]. Consequently, it was more difficult to categorize the activities for persons with MCT and not as accurate as for MCP. So, we decided to merge the activity columns for moderate and vigorous intensity for MCP, which means that all daily activities and arm-crank 20W can be moderate or vigorous depending on % of HR, Borg RPE and speed km/h. The large variations and relative high intensity level for daily activities for MCT has also been reported earlier and that study used % heart rate reserve (HRR) to describe intensity level [49]. Moreover, we explored the use of % of HRR and it showed a lower correlation coefficient (0.6), and the already small span of HR become even smaller for MCT. The result regarding Borg RPE for MCP for different activity and intensity levels is also comparable [13, 38, 46]. For MCT there are fewer comparable studies[38, 48]. However, Goosey-Tolfrey et al[38] found similar levels for light intensity but also had difficulties to distinguish between moderate and vigorous intensity levels using Borg RPE for MCT. Previous research showing cardio metabolic benefits for SCI used a relative intensity between 50–70% of VO_2peak_ or 50–80 HR_peak_ to represent MVPA [50], this indicates that wheeling wheelchair outdoors at 4.8–5.8 km/h or arm-cranking 24-36W at Borg 12–13 could be a way of reaching that intensity level for person with MCP. Hence, for person with MCT wheeling outdoors at 2–6 km/h or arm-crank 10-15W at Borg 13–15, might be more applicable.

### Strengths and limitations

The strengths of this study include the large homogenous groups, for level and severity of injury, and the extensive protocol for several different activities performed by almost all participants. Also, nearly 30% of the study cohorts were women, which enable important and clinically relevant gender comparisons to be made. There was large intra-individual variation for VO_2peak_, affecting the activity-related relative VO_2_ (% of VO_2peak_). This reflects the heterogeneous sample of individuals included in these homogenous subsamples, which means that the included participants have different backgrounds of physical activity. It also shows the complexity of metabolic processes within persons with motor-complete SCI.

The study was limited, however, in that not all activities were represented for both Borg RPE and HR. Additionally, individual PA level may have affected how we categorized the activities. Moreover, variation in mechanical efficiency between the more standardized arm-cranking and the more variable wheeled mobility may have influenced the variation in relative intensity level of performance between these two different modes of activity. Unfortunately, we were not able to compare mechanical efficiency between the wheeled mobility and arm-cranking, as valid measurements of the power output. The results apply only to non-elite athletes within the same limits of BMI and levels of injury and injury severity. Another limitation that could influence the result is that nerve roots projecting from T7-T10 innervate the adrenal gland, and this might contribute to varying degrees of catecholamine spillover during the task. This could contaminate the observed heart-rate responses even when they were standardized to a peak performance[51].

## Conclusions

Using current classification systems, this study has described 11 standardized activities using percentage of VO_2peak_ and categorized them into three intensity levels: light, moderate and vigorous. This translation of the main results (Table 3) is highly relevant as it enables rehab professionals to use clinically accessible methods such as HR-monitoring and Borg RPE to describe intensity levels. This provides tools for better guidance of persons with SCI seeking to meet the desirable, recommended target PA levels.

## Abbreviations

MCP: Motor complete paraplegia
MCT: Motor complete tetraplegia
SCI: Spinal cord injury
PA: Physical Activity
VO_2_: Oxygen consumption
VO_2peak_: Peak oxygen consumption
HR: Heart rate
HR_peak_: Peak Heart rate
RPE: Perceived rate of excretion
C: Cervical
T: Thoracic
AIS: American spinal injury association impairment scale
Penn: Spasm frequency scale
Rpm: Revolutions per minute
Kg: Kilogram
RQ/RER: Respiratory quotient
NEPA: Non-exercise physical activities
BMI: Body mass index
VIF: Variance inflation factor
IQR: Inter quartile range
ACSM: American college of sports and medicine
ml: milliliter
min: minutes
